# Effects of Thymol and Carvacrol, Constituents of *Thymus vulgaris* L. Essential Oil, on the Inflammatory Response

**DOI:** 10.1155/2012/657026

**Published:** 2012-07-05

**Authors:** Fernanda Carolina Fachini-Queiroz, Raquel Kummer, Camila Fernanda Estevão-Silva, Maria Dalva de Barros Carvalho, Joice Maria Cunha, Renata Grespan, Ciomar Aparecida Bersani-Amado, Roberto Kenji Nakamura Cuman

**Affiliations:** ^1^Department of Pharmacology and Therapeutic, State University of Maringá, 870020-900 Maringá, PR, Brazil; ^2^Department of Medicine, State University of Maringá, 870020-900 Maringá, PR, Brazil; ^3^Department of Pharmacology, Federal University of Paraná, 80060-000 Curitiba, PR, Brazil

## Abstract

Thyme (*Thymus vulgaris* L., Lamiaceae) is an aromatic and medicinal plant that has been used in folk medicine, phytopharmaceutical preparations, food preservatives, and as an aromatic ingredient. The effect of *Thymus vulgaris* essential oil (TEO) and its isolated constituents thymol and cavacrol (CVL) were studied in the following experimental models: ear edema, carrageenan-induced pleurisy, and chemotaxis *in vitro*. In the pleurisy model, TEO, CVL, and thymol significantly inhibited inflammatory edema. However, only TEO and CVL inhibited leukocyte migration. In the *in vitro* chemotaxis experiment, CVL inhibited leukocyte migration, whereas thymol exerted a potent chemoattractant effect. In the ear edema model, CVL (10 mg/ear), applied topically, reduced edema formation, exerting a topical anti-inflammatory effect. Thymol did not reduce edema formation but rather presented an irritative response, probably dependent on histamine and prostanoid release. Our data suggest that the antiinflammatory effects of TEO and CVL are attributable to the inhibition of inflammatory edema and leukocyte migration.

## 1. Introduction 

Thyme (*Thymus vulgaris* L., Lamiaceae), a small subshrub native to the western Mediterranean region of Europe, has a long history of use and is a chemically variable species [1]. In folk medicine, some *Thymus* spp. are used for their antihelminthic, expectorant, antiseptic, antispasmodic, antimicrobial, antifungal, antioxidative, antivirotic, carminative, sedative, and diaphoretic effects. They are usually administered by infusion or are used externally in baths to cure rheumatic and skin disease [2, 3]. Reports indicate that the volatile oils of thyme are among the main essential oils used in the food industry and in cosmetics as preservatives and antioxidants [1].

*Thymus vulgaris* essential oil (TEO) is a mixture of monoterpenes. The main compounds of this oil are the natural terpenoid thymol and its phenol isomer carvacrol (CVL) [4, 5], which have antioxidative, antimicrobial, antitussive, expectorant, antispasmodic, and antibacterial effects [6–10]. Terpenoids, flavonoid aglycones, flavonoids glycosides, and phenolic acids were also found in *Thymus* spp. [11].

Several studies have been performed with plant extracts [4, 12, 13], but few studies have evaluated the effects of TEO and its isolated constituents in the inflammatory response. In the present study, the effects of TEO and its isolated components thymol and CVL were studied in experimental models of ear edema, carrageenan-induced pleurisy, and chemotaxis *in vitro*.

## 2. Materials and Methods

### 2.1. Plant Material and Isolation of the Essential Oil

#### 2.1.1. Plant Material

 The fresh leaves of *Thymus vulgaris* L. were collected from the Profa Irenice Silva Medicinal Plant Garden on the campus of the State University of Maringá, Paraná, Brazil. The leaves were identified and authenticated by botanist Maria Aparecida Sert. A voucher specimen was deposited in the Herbarium of the Department of Botany, State University of Maringá (no. 11329). 

#### 2.1.2. Isolation of the Essential Oil

The leaves of *Thymus vulgaris* were extracted by conventional steam distillation using a Clevenger-type apparatus for 2 h. The obtained essential oil was dried over sodium sulphate and stored at 4°C in dark vials until tested. The yield of TEO was 1.76% v/w. Thymol and CVL were isolated from TEO as fractions of hydrodistillated oil. 

### 2.2. Analysis of the Essential Oil and Compound Identification

#### 2.2.1. Gas Chromatography-Mass Spectrometry

 Gas chromatography (GC) was performed with a Thermo Electron Corporation Focus GC model under the following conditions: DB-5 capillary column (30 m × 0.32 mm, and 0.50 mm); column temperature, 60°C (1 min) to 180°C at 3°C/min; injector temperature, 220°C; detector temperature, 220°C; split ratio, 1 : 10; carrier gas, He; flow rate, 1.0 mL/min. The volume injected (1 *μ*L) was diluted in chloroform (1 : 10). The GC/mass spectrometry (MS) analysis was performed with a Quadrupole mass spectrometer (DSQ II model, Thermo Electron Corporation) that operated at 70 V. The identification of the individual compounds was based on comparisons of their GC retention indices (RI) on an apolar column and comparisons with the mass spectra of authentic standards purchased from Sigma-Aldrich and literature data [14]. The retention indices (RI) were obtained with reference to *n*-alkane C_7_H_16_–C_44_H_90_ series (supelco-Bellefonte USA, UK).

#### 2.2.2. Nuclear Magnetic Resonance

The Nuclear Magnetic Resonance (NMR) was used to prove the chemical structure of the essential oil constituents identified by CG-MS. ^1^H (300.06 MHz) and ^13^C nuclear magnetic resonance (NMR; 75.45 MHz) spectra were recorded in a deuterated chloroform (CDCl_3_) solution using a Mercury-300BB spectrometer, with *δ* (ppm) and spectra referenced to CDCl_3_ (*δ* 7.27 for ^1^H and *δ* 77.00 for ^13^C) as the internal standard. 

### 2.3. Animals

Male Wistar rats (weighing 180–220 g) and male Swiss mice (weighing 25–30 g) were provided by the Central Animal House of the State University of Maringá. The animals were housed at 22 ± 2°C under a 12 h/12 h light/dark cycle. Prior to the experiments, the animals were fasted overnight, with water provided ad libitum. The experimental protocols were approved by the Ethical Committee in Animal Experimentation of the State University of Maringá (CEAE/UEM 066/2010).

### 2.4. Bioassays for Cytotoxic Activity

The MTT (3-[4,5-dimethylthiazol-2-yl]-2,5-diphenyl-2H-tetrazolium bromide) assay is based on the mitochondrial enzyme reduction of tetrazolium dye that detects and determines cell viability. The leukocytes were obtained from the peritoneal cavity of mice 4 h after zymosan injection (1 mg/cavity, i.p.). Briefly, the cells (5 × 10^5^ cells/well) were exposed to CVL (1, 3, 10, 30, and 90 *μ*g/mL), thymol (1.5, 15, and 150 *μ*g/mL, dissolved in dimethyl sulfoxide [DMSO]), or TEO (1, 3, 10, 30, and 90 *μ*g/mL) for 90 min at 37°C in 5% CO_2_. A volume of 10 *μ*L of MTT (5 mg/mL; Sigma) was added to each well. After 2 h, 150 *μ*L of the supernatant was removed, and 100 *μ*L DMSO was added to each well. The cells were incubated at 25°C for an additional 10 min, and absorbance was measured using a Biochrom Asys Expert plus microplate reader (Asys) at a wavelength of 540 nm. The values of the blank wells were subtracted from each well of treated and control cells. The percentage of viability was determined by the following formula:

(1) %viable  cells:(Absorbance of the treated cells−Absorbance of the blank)/(Absorbance of the control−Absorbance of the blank)×100.

### 2.5. Acute Toxicity Test

Fasted mice were orally treated with TEO. The doses progressively increased to determine the dose necessary to produce lethality in 50% of the animals (LD50). The mice were observed for 7 days following the treatments. Food and water were provided ad libitum throughout the experiment. The number of mice that died within the study period was noted for each group. The LD50 was calculated according to the literature [15]. An equivalent dose of vehicle was administered to the control group.

### 2.6. Carrageenan-Induced Pleurisy in Rats

This test was performed according to the technique described by Vinegar et al. [16]. The animals were orally pretreated with TEO (250, 500, or 750 mg/kg), CVL (100, 200, or 400 mg/kg), and thymol (100, 200, or 400 mg/kg). Indomethacin (5 mg/kg) and celecoxib (10 mg/kg) were used as standard drugs. Control rats received only water. One hour later, all of the animals, with the exception of the normal group, received an intrapleural injection of carrageenan (200 *μ*g/animal). Four hours later, the animals were euthanized, and the pleural exudate was collected. The volume was determined, and the pleural cavity was washed with 2.0 mL phosphate-buffered saline (PBS) that contained EDTA. The exudate volume was measured, and a 50 *μ*L aliquot was used to determine the number of leukocytes in a Neubauer chamber. For total leukocyte count, red blood cells were lysed by adding Turk's solution. For differential cell counting, the fluid was centrifuged at 2500 rotations per minute for 10 min. Exudate smears were prepared, dried, fixed, and stained with May-Grunwald-Giemsa. The numbers of mononuclear and polymorphonuclear leukocytes in the exudate were determined by optical microscopy, with 100 cells counted per slide. The results are expressed as mean ± SEM.

### 2.7. *In Vitro* Chemotaxis

To evaluate the effects of CVL and thymol on chemotaxis, leukocytes were obtained from the peritoneal cavity of mice 4 h after zymozan injection (1 mg/cavity, i.p). The cell number was adjusted to 1 × 10^6^ cells/mL in RPMI medium containing 0.1% bovine serum albumin (BSA). The chemotaxis assay was performed using a 48-well microchemotaxis plate (Neuro Probe), in which the chambers were separated by a polyvinylpyrrolidone-free polycarbonate membrane (5 *μ*m pore size). The chemoattractants *N*-formyl methionyl leucyl phenylalanine (fMLP; 10^−6^ M) and leukotriene B4 (LTB4; 10^−8^ M) and a negative control (RPMI 1640) were placed in the lower chamber. A leukocyte suspension (1 × 10^6^ cells/mL) pretreated with thymol (0.3, 1, 3, 10, 30, or 90 *μ*g/mL) and CVL (0.3, 1, 3, 10, 30, or 90 *μ*g/mL) for 30 min was then placed in the upper chamber. The cells were allowed to migrate into the membrane for 1 h at 37°C in 5% CO_2_. Following incubation, the membrane was washed in PBS, fixed in methanol, and stained with Instant Prov. The membrane area of each well was scored using light microscopy to count the intact cells present in five random fields. The results are expressed as the mean number of leukocytes per field and are representative of three separate experiments.

To evaluate the chemoattractant effect of thymol on leukocyte chemotaxis, thymol was tested at concentrations of 1.5, 15, and 150 *μ*g/mL. The cells were obtained from the peritoneal cavity as described above. Thymol or RPMI 1640 were placed in the lower chamber. The leukocyte suspension (1 × 10^6^ cells/mL) was placed in the upper chamber, and the chemotaxis assay was performed as described above.

### 2.8. Topical Ear Edema

Cutaneous inflammation was induced by the application of 5% croton oil (10 *μ*L) in acetone (vehicle) to the inner surface of the right ear in mice. The left ear received an equal volume of vehicle. CVL (10, 20, and 40 mg/ear), thymol (10 mg/ear), indomethacin (0.5 mg/ear), dexamethasone (0.1 mg/ear), and vehicle were topically applied to the right ear 1 h before croton oil application. Four hours after the inflammatory stimulation, the mice were sacrificed, and a plug (7 mm diameter) was removed from both the treated and untreated ears (*n* = 10). Edema was measured as the weight difference between the two plugs. The data are expressed as the mean ± SEM weight of the ears.

Thymol as a topical irritative was also tested. Ten microliters of thymol was applied to the right ears of mice, and the left ears received an equal volume of vehicle (acetone). The animals were treated with indomethacin (5 mg/kg, orally) or promethazine (5 mg/kg, i.p.) 60 min before the thymol application. Croton oil was used as a positive control. Ear edema was determined 30, 60, 120, 180, and 240 min after the inflammatory stimulation and is expressed as the increase in ear thickness measured with an electronic micrometer (Digimess) before and after the induction of the inflammatory response. The micrometer was applied near to the tip of the ear just distal to the cartilaginous ridges, and the thickness was determined in micrometers. To minimize the variations caused by using this technique, a single investigator made the measurements throughout all of the experiments. The data are expressed as the mean ± SEM ear measurements.

### 2.9. Myeloperoxidase Activity

Myeloperoxidase (MPO) activity was assayed in the supernatant of homogenates of the ear sections (untreated controls and animals treated with CVL, thymol, and 0.1 mg dexamethasone) [17]. The ears were placed in 50 mM potassium phosphate buffer (pH 6.0) that contained 0.5% hexadecyl trimethyl ammonium bromide (1 mL/50 mg of tissue; Sigma, St. Louis, MO, USA) in a Potter homogenizer. The homogenate was shaken in a vortex mixer and centrifuged for 5 min. Ten microliters of the supernatant was added to each well in triplicate in a 96-well microplate. Two hundred microliters of the buffer solution that contained 16.7 mg *O*-dianisidine dihydrochloride (Sigma), 90 mL double-distilled water, 10 mL potassium phosphate buffer, and 50 *μ*L of 1% H_2_O_2_ was added. The enzyme reaction was stopped by the addition of sodium acetate. Enzyme activity was determined by the absorbance measured at 460 nm using a microplate spectrophotometer (Spectra Max Plus).

### 2.10. Statistical Analysis

The data are expressed as the mean ± SEM for each group. The data were statistically analyzed using one-way variance analysis followed by Tukey's test. Differences were considered significant at *P* < 0.05.

## 3. Results and Discussion

**Figure 1:**
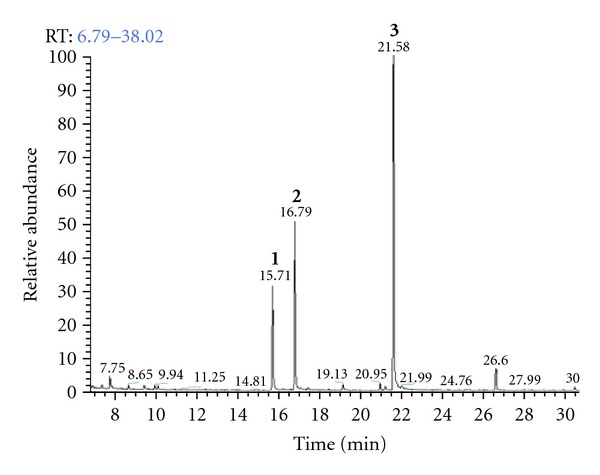
GC chromatogram of *Thymus vulgaris *L. essential oil. Percentage data were obtained by gas chromatography-mass spectrometry (GC-MS). Peak (1)  *endo*-Borneol (14.3%); (2) *α*-Terpineol (22.9%); (3)  Carvacrol (45.5%).

The essential oils obtained from the leaves of *Thymus* are rich in monoterpene phenols, especially thymol and CVL [5]. The chemical composition of TEO was investigated using GC-MS and NMR. The results of the GC-MS analysis (Figure 1) showed a predominance of CVL (45.5%), *α*-terpineol (22.9%), and *endo*-Borneol (14.3%). The percentages of the major components and their retention indices are summarized in Table 1.

In the cell viability assay, the treatments were tested at different concentrations. TEO at concentrations of 1, 3, 10, 30, and 90 *μ*g/mL showed cell viability of 88%, 82%, 90%, 92%, and 75%, respectively. CVL at concentrations of 1, 3, 10, 30, and 90 *μ*g/mL showed cell viability of 90%, 90%, 90%, 88%, and 87%, respectively. Thymol at concentrations of 1.5, 15, and 150 *μ*g/mL showed cell viability of 83%, 97%, and 95%, respectively. Our data indicated that TEO, CVL, and thymol did not present *in vitro* cytotoxicity at any of the concentrations tested.

In the acute toxicology study, TEO was tested orally at doses of 2000 mg/kg, 3000 mg/kg, and 4000 mg/kg. The LD50 value of TEO was 4000 mg/kg. All of the doses used in the present study were lower than the observed LD50 values. Consequently, no apparent behavioral side effects were observed in the animals during our studies. The high LD50 values also suggest that TEO is relatively safe and nontoxic. Therefore, we studied the effects of TEO, CVL, and thymol on the inflammatory response evaluated by antiedematogenic activity and leukocyte migration.

Acute inflammation, typically characterized by redness, swelling, pain, and heat, is one of the most important defense mechanisms against invading pathogens. Lipopolysaccharide (LPS) can active monocytes, neutrophils, and macrophages [18] and induce an oversecretion of various proinflammatory and toxicity-mediating molecules, such as tumor necrosis factor *α* (TNF-*α*), interleukin-6 (IL-6), eicosanoids, and nitric oxide (NO) [19]. Prostaglandins (PGs) and NO are two important proinflammatory mediators. The inhibition of the production of PGs and NO via the inhibition of their synthases (i.e., cyclooxygenase 2 [COX-2] and inducible nitric oxide synthase [iNOS], resp.) has been demonstrated to be beneficial in the treatment of inflammatory disease [20]. Anti-inflammatory drugs, such as steroids and nonsteroidal anti-inflammatory drugs (NSAIDs), have numerous adverse side effects, such as gastrointestinal discomfort, the inhibition of platelet aggregation, and liver and kidney toxicity [21]. Therefore, the search for natural products with fewer side effects has been increasingly important.

Different mechanisms are well known to be involved in the genesis of inflammatory reactions. The development of inflammatory edema induced by carrageenan is characterized by an initial stage (1-2 h) and is dependent on the release of histamine, serotonin, and bradykinin, followed by a later stage (3-4 h) that is maintained principally by the release of kinins, lysozymes, and prostanoids [22, 23]. Eicosanoids promote the chemotaxis of neutrophils, and they induce the biosynthesis of elastase, collagenase, and other compounds. These enzymes break down structural proteins into peptides. Consequently, vascular permeability and hydrostatic pressure increase, resulting in edema and the migration of neutrophils to the damaged tissue [24]. COX-2, an inducible enzyme found in activated inflammatory cells, plays a crucial role in cytokine production and prostanoid mediator release. The inhibition of COX-2 protein expression has been used to evaluate the anti-inflammatory effects of compounds *in vivo* and *in vitro* [19, 25, 26]. TNF-*α*, a key mediator of the inflammatory response, stimulates innate immune responses by activating T-cells and macrophages that stimulate the release of other inflammatory cytokines. TNF-*α* is also a mediator of carrageenan-induced inflammation and is able to enhance the further release of kinins and leukotrienes [27]. Nitric oxide has been shown to play an important role in both the regulation of vascular permeability and cell migration induced by proinflammatory agents, including carrageenan [28, 29].

The pleurisy model is used to screen anti-inflammatory drugs. Exudate accumulation in the pleural cavity and leukocyte migration can be evaluated [16]. In the pleurisy model, TEO at doses of 250, 500, and 750 mg/kg significantly reduced inflammatory exudates. At a dose of 750 mg/kg, TEO reduced the number of migrated cells (Table 2). The groups treated with indomethacin and celecoxib exhibited a reduction in inflammatory exudates but not a reduction in leukocyte migration. CVL and thymol significantly reduced the volume of pleural inflammatory exudates by 47.3% and 34.2%, respectively, at a dose of 400 mg/kg. CVL decreased the number of migrated cells at doses of 100, 200, and 400 mg/kg. Thymol, however, was not able to reduce cell migration (Tables 3 and 4). These data indicate that TEO, CVL, and thymol significantly inhibited inflammatory edema, but only TEO and CVL exerted inhibitory effects on leukocyte migration to the injury site. Our data corroborate previous studies that demonstrated the anti-inflammatory effects of different essential oils (i.e., inhibition of inflammatory edema and chemotaxis) [30–34]. CVL acts as a suppressor of COX-2 and activator of peroxisome proliferator-activated receptors [35]. Our data suggest that CVL may inhibit prostanoid release because CVL had effects that were similar to indomethacin and celecoxib (i.e., inhibition of COX-1 and -2) [36] (Table 3).

Polymorphonuclear leukocyte recruitment is an essential factor in the acute inflammatory process, acting as first-line-defense cells in the initiation and resolution phases of this process [37]. In situations in which uncontrolled infiltration of these cells occurs, they can become the main aggressor factor. Under such conditions, pharmacological interventions with drugs that are able to modulate leukocyte recruitment may present an interesting therapeutic possibility.

**Figure 2:**
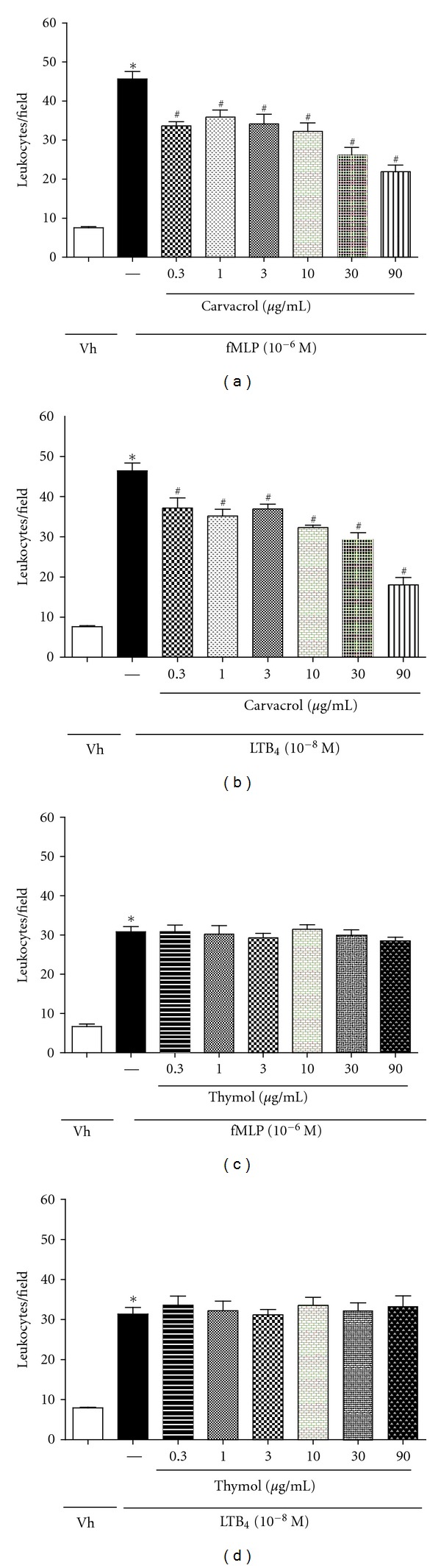
Effect of CVL and thymol on *in vitro* leukocyte chemotaxis. Leukocytes were obtained from zymosan-induced peritonitis (200 *μ*g/cavity) and stimulated with fMLP (10^−6^) or LTB_4_ (10^−8^) after 30 min of CVL (a, b) or thymol (c, d) treatments at doses of 0.3, 1, 3, 10, 30 and 90 *μ*g/mL. Values are mean ± SEM (*n* = 5) and are representative of three independent experiments. *P < 0.05 versus Vh (vehicle), ^#^ *P* < 0.05 versus group of leukocytes stimulated with fMLP or LTB_4_.

**Figure 3:**
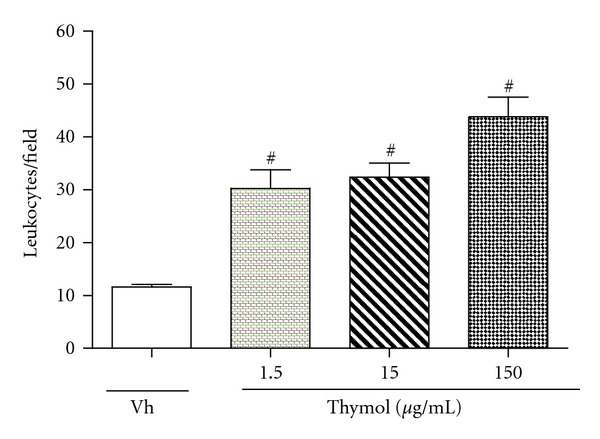
Thymol used as a chemotactic agent in concentrations of 1.5, 15, and 150 *μ*g/mL. Leukocytes were obtained from zymosan-induced peritonitis (200 *μ*g/cavity). Values are mean ± SEM (*n* = 5) and are representative of three independent experiments. ^#^ *P* < 0.05 versus Vh (vehicle).

The present study also evaluated the effects of thymol and CVL at different concentrations on leukocyte chemotaxis *in vitro*. The chemoattractants fMLP (10^−6^ M) and LTB_4_ (10^−8^ M) were used. CVL at doses of 0.3, 1, 3, 10, 30, and 90 *μ*g/mL significantly reduced (*P* < 0.05) neutrophil migration in response to fMLP stimulation (20.07 ± 1.02%, 14.79 ± 1.79%, 25.28 ± 2.51%, 29.48 ± 2.21%, 42.92 ± 2.06%, and 52.23 ± 1.75%, resp.) and LTB_4_ stimulation (19.82 ± 2.50%, 24.81 ± 1.66%, 20.35 ± 1.18%, 30.41 ± 0.61%, 36.44 ± 1.54%, and 61.11 ± 1.82%, resp.; Figures 2(a) and 2(b)). Thymol at doses of 0.3, 1, 3, 10, 30, and 90 *μ*g/mL did not reduce leukocyte migration *in vitro* in response to fMLP and LTB_4_ stimulation (Figures 2(c) and 2(d)). However, thymol at concentrations of 1.5, 15, and 150 *μ*g/mL was a potent chemoattractant agent (Figure 3). CVL and thymol did not affect leukocyte viability at the concentrations tested, suggesting that the direct effects of the treatments on the inhibition of leukocyte chemotaxis did not occur because of the toxic effects that induce cell death.

Only CVL was able to inhibit *in vitro* chemotaxis induced by fMLP and LTB_4_, suggesting that CVL and thymol exert their effects on leukocyte chemotaxis through different mechanisms. Leukotriene is a potent chemotactic agent derived from arachidonic acid [38]. fMLP is a chemotactic agent involved in the release of cytokines. Upon binding to its G-protein-coupled receptor, it activates multiple signaling cascade pathways [39]. These pathways include the mitogen-activated protein kinase (MAPK) and phosphatidylinositol 3-kinase (PI-3K) cascades, which are important for the development of the functional responses of neutrophils in inflammation [40, 41]. Our data suggest that CVL may act by inhibiting cytokines and leukotrienes, and these mediators are likely not involved in the mechanism of action of thymol.

**Figure 4:**
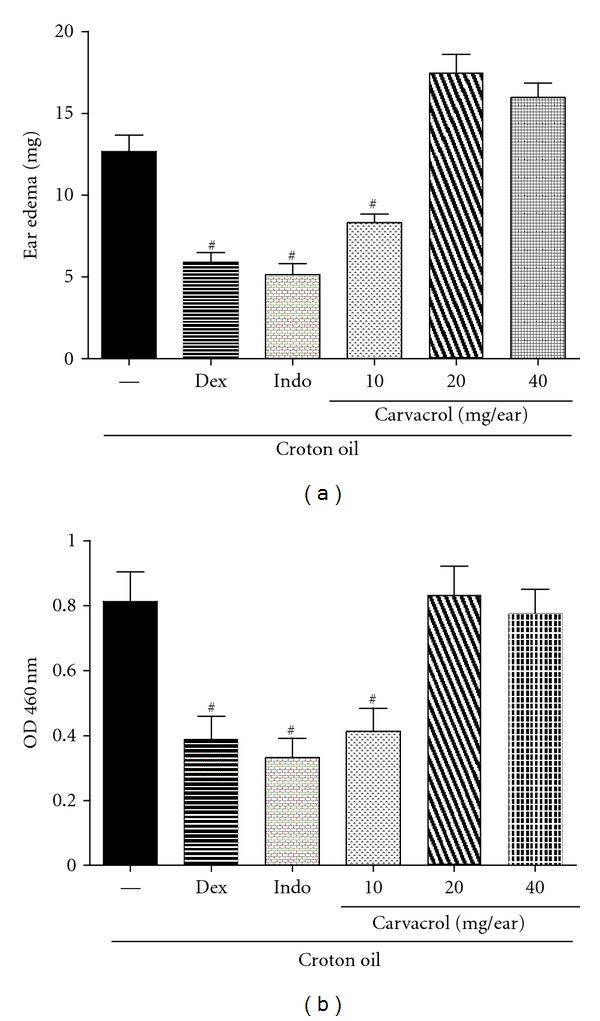
Effect of CVL on ear edema (a) and myeloperoxidase activity (MPO) (b) induced by croton oil in ear tissues from mice. The animals (*n* = 9) were treated topically with carvacrol, indomethacin (Indo), or dexamethasone (Dex) 1 h before croton oil application (10 *μ*L/ear). Dex (0.1 mg/ear) or Indo (0.5 mg/ear) were used as anti-inflammatory drugs (positive control). The right ears received only the vehicle (Basal). Data are mean ± SEM weight of the ears (a) or MPO activity (b), 4 hours after application of croton oil, ^#^ *P* < 0.05, compared to the control group (croton oil) (ANOVA, Tukey's test).

To demonstrate the topical effect of thymol and CVL *in vivo*, we evaluated inflammatory ear edema induced by croton oil. Croton oil is an irritant agent that causes cell damage and activates phospholipase A_2_, which releases arachidonic acid from the cell plasma membrane. From arachidonic acid, the production of prostaglandins by COX-1 and COX-2 and leukotrienes by 5-lipoxygenase occurs. Prostaglandins and leukotrienes are inflammatory mediators involved in edema and leukocyte migration [42]. Croton oil application to the right ear in mice induced an apparent inflammatory response 4 h later. An increase in the weight of the ears was observed. Indomethacin (0.5 mg/ear) and dexamethasone (0.1 mg/ear) significantly inhibited ear edema by 44% and 36%, respectively (*P* < 0.05). CVL did not reduce ear edema at concentrations of 20 and 40 mg/ear, but ear edema was reduced at the lowest concentration (10 mg/ear), similar to the 37.2% reduction observed with dexamethasone (*P* < 0.05; Figure 4(a)).

The present study showed that CVL at 10 mg/ear has an antiedematogenic effect when administered topically, similar to the observations with dexamethasone and indomethacin. In this experimental model, these treatments inhibited both fluid extravasation and cellular influx, indirectly reflected by a reduction in MPO activity. The enzyme MPO is found in the azurophilic granules of neutrophils and other cells of myeloid origin and is considered a marker of polymorphonuclear leukocyte influx into inflamed tissues. Therefore, MPO inhibition may result in an anti-inflammatory effect [23]. CVL (10 mg/ear), indomethacin (0.5 mg/ear), and dexamethasone (0.1 mg/ear) significantly inhibited the activity of this enzyme (43.8%, 52.0%, and 38.3%, resp.; *P* < 0.05; Figure 4(b)). Our data showed that CVL effectively inhibited chemotaxis *in vitro* but also had antiedematogenic and antichemotactic effects *in vivo* (pleurisy test) when administered systemically.

**Figure 5:**
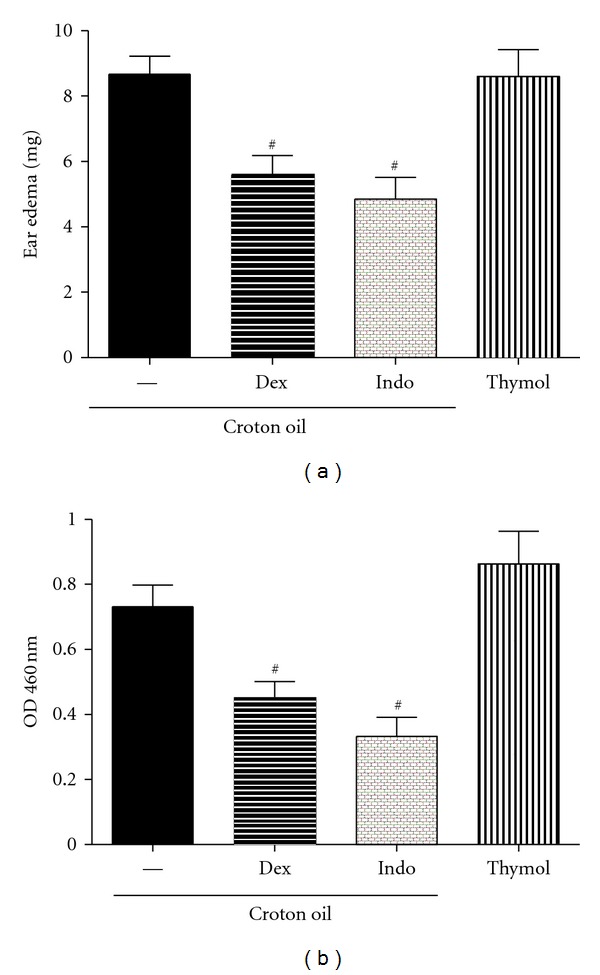
Effect of thymol on ear edema (a) and myeloperoxidase activity (MPO) (b) in ear tissues from mice. The animals (*n* = 9) were treated topically with indomethacin (Indo) or dexamethasone (Dex) 1 h before croton oil application (10 *μ*L/ear). The group treated with thymol did not received the croton oil. Dex (0.1 mg/ear) and Indo (0.5 mg/ear) were used as anti-inflammatory drugs (positive control). The right ears received only the vehicle. Data are mean ± SEM weight of the ears (a) or MPO activity (b), 4 hours after application of croton oil or thymol. ^#^ *P* < 0.05, compared to the control group (croton oil) (ANOVA, Tukey's test).

**Figure 6:**
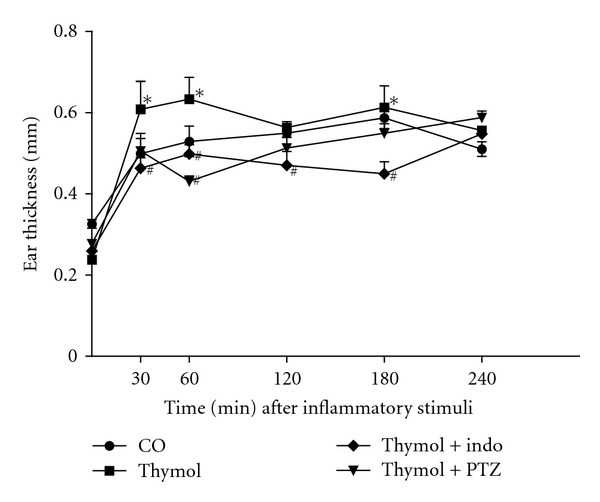
Ear edema induced by thymol (10 mg/ear). The animals (*n* = 8) were treated with indomethacin (Indo) (5 mg/Kg, v.o.) or promethazine (PTZ) (5 mg/Kg, i.p.) 60 minutes beforethe thymol application. The croton oil (CO) was used for positive control. The ear edema was determined in 30, 60, 120, 180, and 240 minutes after inflammatory stimuli. Data are mean ± SEM of the ear thickness, ^#^ *P* < 0.05 compared to thymol group. *P < 0.05 compared to the positive control (ANOVA, Tukey's test).

As shown in Figure 5, the ear edema formation intensities were similar between croton oil and thymol, suggesting similar responses to both irritative agents. Ear edema induced by croton oil involves the activation of phospholipase A_2_ and biosynthesis of prostaglandins and leukotrienes [43, 44]. To compare the probable irritative mechanism of the topical effect of thymol and croton oil, the following experiments were performed. The animals were treated with indomethacin (5 mg/kg, p.o.) and promethazine (5 mg/mL, i.p.) 60 min before the application of thymol. Croton oil was used as a positive control. To evaluate the participation of histamine in this inflammatory response, promethazine was used as an antihistaminic reference drug. Ear edema was determined 30, 60, 120, 180, and 240 min after the inflammatory stimulation. The time-course analysis of thymol revealed an increase in edema volume at 60 min (Figure 6). This response could be attributable to the release of different autacoids, including histamine. Essential oils and their isolated compounds can promote the release of histamine and other mediators, acting as irritative agents [45]. Indomethacin treatment effectively reduced the formation of ear edema induced by thymol, whereas promethazine treatment only inhibited the early stage of edema. Our results indicate that the edematogenic response of thymol is partially dependent on histamine and prostanoids. Therefore, our results suggest that thymol has an irritative effect, similar to the effect observed with croton oil.

Thus, our results are consistent with the literature and showed that TEO has anti-inflammatory effects *in vivo*. However, the isolated compounds thymol and CVL showed antagonist effects. Thymol has an irritative effect that likely involves histamine, prostanoids, and other inflammatory mediators. CVL may be the compound responsible for the anti-inflammatory effects of TEO, demonstrated by chemotaxis *in vitro*. The present study contributes to the growing evidence of the anti-inflammatory effects of natural products. Overall, our data support the hypothesis that the inhibitory effect of CVL on leukocyte migration contributes to its anti-inflammatory action, in addition to the irritant effect of thymol.

## Figures and Tables

**Table 1:** Percentual chemical composition of *Thymus vulgaris* leaves essential oil.

Retention time	Retention index^a^	Compound	Percentual (%)	Identification
6.8	945	Solvente	—	MS^b^
7.8	960	*α*-Pinene	1.9	MS, NMR^d^
8.7	1022	*p*-Cymene	0.6	MS, NMR
9.9	1022	Limonene	0.6	MS, NMR
10.1	1028	*γ*-Terpinene	1.1	MS, NMR
11.3	1056	Linalool	0.2	MS, NMR
14.8	1143	Camphor	0.1	MS, NMR
15.7	1163	*endo*-Borneol	14.3	MS, NMR
16.2	1175	4-Terpineol	0.7	MS, NMR
16.8	1187	*α*-Terpineol	22.9	MS, NMR
19.1	1240^c^	Carvacrol methyl ether	1.3	MS, NMR
21.0	1289^c^	Thymol	0.9	MS, NMR
21.6	1297^c^	Carvacrol	45.5	MS, NMR
24.8	1417	Geranyl acetate	0.3	MS, NMR
26.6	1417	Caryophyllene	3.2	MS, NMR
30.8	1580	Unknown	0.8	MS, NMR
33.2	1580	Caryophyllene oxide	2.9	MS, NMR
35.3	1637	Germacrene-D	1.8	MS, NMR
38.0	1708	Unknown	0.1	MS, NMR

Total identified			99.2	

^a^RI: Retention index relative to a homologous series of *n*-alkanes on the DB-5 capillary column.

^b^Mass spectrometry.

^c^Calculated considering retention time of 22.65 minutes.

^d^Nuclear magnetic resonance.

**Table 2:** Effect of *Thymus vulgaris* essential oil (TEO) treatment on exudate volume and leukocytes number 4 hours after carrageenan injection (200 *μ*g/pleural cavity) in rats.

Group	Exudate volume (mL)	Inhibition (%)		(cells/mm^3^) × 10^3^	
			Total leukocytes	MN	PMN
Normal	0.16 ± 0.01		6700 ± 450	1800 ± 160	4900 ± 390
Control	0.76 ± 0.03^b^		55650 ± 1860^b^	8831 ± 644^b^	46819 ± 1399^b^
Indomethacin (5 mg/kg)	0.33 ± 0.02^a,b^	56.7	60250 ± 7600^b^	10162 ± 1137^b^	50088 ± 6989^b^
Celecoxib (10 mg/kg)	0.43 ± 0.03^a,b^	43.3	42350 ± 3536^b^	6972 ± 1047^b^	35378 ± 3774^b^
TEO					
250 mg/kg	0.62 ± 0.02^a,b^	18.4	52696 ± 2558^b^	10786 ± 1156^b^	41910 ± 2646^b^
500 mg/kg	0.61 ± 0.03^a,b^	19.7	54729 ± 3090^b^	7457 ± 924.9^b^	47272 ± 2354^b^
750 mg/kg	0.53 ± 0.04^a,b^	30.2	41417 ± 2125^a,b^	5873 ± 415.7^b^	35543 ± 1801^a,b^

Data are mean ± SEM of 8–10 animals/group. Indomethacin and celecoxib administered orally were used as reference anti-inflammatory drugs (positive controls). Normal: animals that received injection of saline in the cavity (normal group), Control: animals that received injection of carrageenan in the cavity (negative control). MN: mononuclears cells. PMN: polimorphonuclears cells. ^a^ *P* < 0.05 compared to control group; ^b^ *P* < 0.05 compared to normal group (ANOVA, Tukey's test).

**Table 3:** Effect of carvacrol treatment on exudate volume and leukocytes number 4 hours after carrageenan injection (200 *μ*g/pleural cavity) in rats.

Group	Exsudate volume (mL)	Inhibition (%)		(cells/mm^3^) × 10^3^	
			Total leukocytes	MN	PMN
Normal	0.16 ± 0.01		6700 ± 450	1800 ± 160	4900 ± 390
Control	0.76 ± 0.03^b^		55650 ± 1860^b^	8831 ± 644^b^	46819 ± 1399^b^
Indomethacin (5 mg/kg)	0.33 ± 0.02^a,b^	56.7	60250 ± 7600^b^	10162 ± 1137^b ^	50088 ± 6989^b ^
Celecoxib (10 mg/kg)	0.43 ± 0.03^a,b^	43.3	42350 ± 3536^b^	6972 ± 1047^b^	35378 ± 3774^b^
Carvacrol					
100 mg/kg	0.68 ± 0.01^b^	—	32250 ± 3256^a,b^	4522 ± 635.3^b^	27728 ± 2696^b^
200 mg/kg	0.65 ± 0.02^b^	—	38625 ± 2617^a,b^	5544 ± 430.9^b^	33081 ± 2171^b^
400 mg/kg	0.40 ± 0.05^a,b^	47.3	33583 ± 2548^a,b^	4914 ± 353.8^b^	28669 ± 2211^a,b^

Data are mean ± SEM of 8–10 animals/group. Indomethacin and celecoxib administered orally were used as reference anti-inflammatory drugs (positive controls). Normal: animals that received injection of saline in the cavity (normal group), Control: animals that received injection of carrageenan in the cavity (negative control). MN: mononuclears cells. PMN: polimorphonuclears cells. ^a^ *P* < 0.05 compared to control group; ^b^ *P* < 0.05 compared to normal group (ANOVA, Tukey's test).

**Table 4:** Effect of thymol treatment on exudate volume and leukocytes number 4 hours after carrageenan injection (200 *μ*g/pleural cavity) in rats.

Group	Exsudate volume (mL)	Inhibition (%)		(cells/mm^3^) × 10^3^	
			Total leukocytes	MN	PMN
Normal	0.16 ± 0.01		6700 ± 450	1800 ± 160	4900 ± 390
Control	0.76 ± 0.03		55650 ± 1860^b^	8831 ± 644^b^	46819 ± 1399^b^
Indomethacin (5 mg/kg)	0.33 ± 0.02^a^	56.7	60250 ± 7600^b^	10162 ± 1137^b^	50088 ± 6989^b^
Celecoxib (10 mg/kg)	0.43 ± 0.03^a^	43.3	42350 ± 3536^b^	6972 ± 1047^b^	35378 ± 3774^b^
Thymol					
100 mg/kg	0.73 ± 0.06	—	42306 ± 2429^b^	5563 ± 403.6^b^	36742 ± 2216^b^
200 mg/kg	0.65 ± 0.03	—	46900 ± 2931^b^	6609 ± 683.8^b^	40290 ± 2357^b^
400 mg/kg	0.54 ± 0.05^a^	34.2	47600 ± 2987^b^	6286 ± 380.3^b^	41313 ± 2979^b^

Data are mean ± SEM of 8–10 animals/group. Indomethacin and celecoxib administered orally were used as reference anti-inflammatory drugs (positive controls). Normal: animals that received injection of saline in the cavity (normal group), Control: animals that received injection of carrageenan in the cavity (negative control). MN: mononuclears cells. PMN: polimorphonuclears cells. ^a^ *P* < 0.05 compared to control group; ^b^ *P* < 0.05 compared to normal group (ANOVA, Tukey's test).
